# Diagnostic yield of post-bronchoscopy sputum for diagnosing pauci-bacillary pulmonary tuberculosis

**DOI:** 10.1080/07853890.2021.1908587

**Published:** 2021-05-08

**Authors:** Joung Ha Park, Kyung-Wook Jo, Tae Sun Shim, Sung-Han Kim

**Affiliations:** a Departments of Infectious Diseases, Asan Medical Center, University of Ulsan College of Medicine, Seoul, Republic of Korea; b Pulmonology and Critical Care Medicine, Asan Medical Center, University of Ulsan College of Medicine, Seoul, Republic of Korea

**Keywords:** Post-bronchoscopy sputum, pulmonary tuberculosis, pauci-bacillary

## Abstract

**Background:**

A few studies have mentioned that post-bronchoscopy sputum (PBS) could improve the diagnostic yield in pauci-bacillary pulmonary tuberculosis (PTB). Therefore, we evaluated the diagnostic yield of PBS for diagnosing pauci-bacillaryPTB.

**Methods:**

Clinical data of immunocompromised adult patients with pauci-bacillary PTB were retrospectively retrieved at a tertiary hospital in Seoul, South Korea over a 5-year period. We analyzed patients who underwent bronchoscopy examinations for diagnosing pauci-bacillary PTB.

**Results:**

Ninety patients were finally analyzed. Of these patients, 76 patients were tested with PBS. Six (8%) of these patients had positive results on AFB smear of PBS alone. Additionally, 52 patients (68%) had positive results on mycobacterial culture and 12 (16%) had positive results on mycobacterial culture of PBS exclusively. Therefore, in this study population, a total of 18 patients (20%) were finally diagnosed as having PTB with PBS results only, even though AFB smear microscopy and culture of other specimens had negative results.

**Conclusions:**

PBS could improve the diagnostic yield by 20% when diagnosing pauci-bacillary PTB. In addition, about 8% of the patients could be diagnosed rapidly because of AFB smear microscopy positivity for PBS. Therefore, PBS use should be considered as a complementary diagnostic approach in patients with suspected pauci-bacillary PTB.

## Introduction

Atypical presentations are common in immunocompromised patients with tuberculosis (TB), and the diagnosis and anti-TB treatment are sometimes delayed until mycobacterial culture is available in about half of immunocompromised patients with pauci-bacillary TB [1]. Thus, the diagnosis of TB in these patients would be confirmed after the results of mycobacterial culture or molecular diagnostic tests, although these tests could also reveal negative results. Therefore, the efforts to increase the diagnostic yield of mycobacterial culture are important. In this clinical situation, bronchoscopy examinations with bronchial aspiration or Broncho alveolar lavage (BAL) are usually performed in these patients with suspected pauci-bacillary pulmonary TB. However, when rapid diagnostic tests of bronchial aspiration or BAL also reveal negative results, further microbiologic tests of post-bronchoscopy sputum (PBS) occasionally exhibit positive results. There are few studies on whether PBS improves the diagnostic performance for pauci-bacillary pulmonary TB [2–4]. Therefore, we evaluated the diagnostic yield of PBS for diagnosing pauci-bacillary pulmonary TB in immunocompromised patients.

## Materials and methods

### Study population and study design

**Figure 1.**
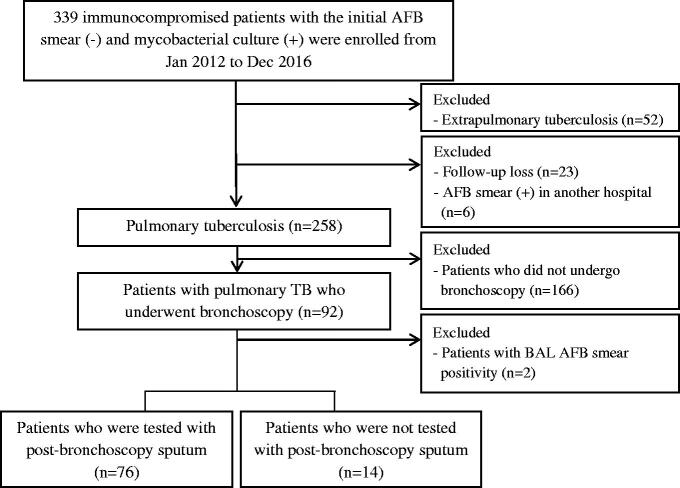
Schematic flow chart of the study.

This retrospective study was performed at Asan Medical Centre, a 2,700-bed tertiary care teaching hospital in Seoul, South Korea, which is a country with an intermediate TB burden (annual TB incidence in 2016 of 63.2 per 100,000 population) [5] and low human immunodeficiency virus (HIV) burden. Clinical data of all immunocompromised adult patients (aged ≥18 years) with pauci-bacillary TB were retrospectively retrieved from January 2012 to December 2016. We excluded patients with extra pulmonary TB or those who were not followed up or transferred from other hospitals with positive results on AFB smear microscopy at those hospitals. Subsequently, we only analyzed patients who underwent bronchoscopy examinations with bronchial washing or BAL for diagnosing pulmonary TB (Figure 1). The study protocol was approved by the Institutional Review Board of our hospital. Informed consent was waived because of the retrospective nature of this study.

### Definition

Pauci-bacillary pulmonary TB was defined as microbiologically confirmed pulmonary TB with negative results on AFB smear of the initial respiratory specimens (sputum, bronchial aspirates, and BAL fluid). Immunocompromised patients were defined as patients with predisposing factors for TB, such as HIV infection, malignancy, liver cirrhosis, chronic renal failure, and transplantation status, or receipt of immunosuppressive treatment [6–8].

### Mycobacterial culture and Xpert

For culture identification of mycobacteria, respiratory specimens were inoculated in liquid (Bactec MGIT 960) and solid (Ogawa media) culture media and cultured for at least 8 weeks. And Xpert was performed following the manufacturer’s protocol and previous reports [9,10].

### Statistical analysis

Statistical calculations including proportion and mean with standard deviation were performed using IBM SPSS Statistics for Windows, version 21.0 (IBM Corp., Armonk, NY, USA). This is a proof-of-concept study for pilot testing, so we used convenient sample size.

## Results

**Figure 2.**
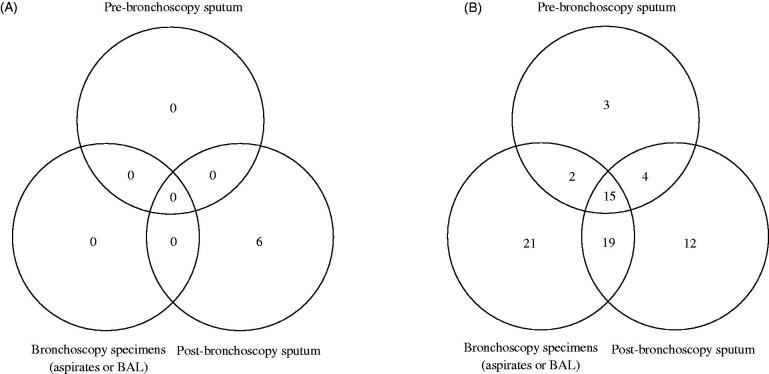
(A) AFB smear-positive results of respiratory specimens (*n* = 76). Six patients had positive results of AFB smear of post-bronchoscopy sputum only. The remaining 70 patients had negative results of AFB smears of all respiratory specimens. (B) Mycobacterial culture-positive results of respiratory specimens (*n* = 76).

Amonga total of 92 immunocompromised adult patients with pauci-bacillary pulmonary TB, 2 patients were excluded owing to AFB smear positivity for bronchoscopy specimens after the initial negative results for expectorated sputum. The remaining 90 patients were finally analyzed (Figure 1). The baseline demographic characteristics of all patients are shown in Table 1. Of these 90 patients, 43 patients were tested with pre-bronchoscopy sputum and 76 patients were tested with PBS. The comparisons of positive results on AFB smear and culture among various respiratory specimens (pre-bronchoscopy sputum, bronchoscopy specimens, and PBS) are shown in Figure 2 (Venn diagram). While pre-bronchoscopy sputum and bronchoscopy specimens exhibited negative AFB smear results in all patients, PBS samples revealed positive AFB smear results in 6 (8%) of the 76 patients. In addition, 64 (84%) patients had positive results on mycobacterial culture of pre-bronchoscopy sputum and bronchoscopy specimens, and 12 patients (16%) had positive results on mycobacterial culture of PBS exclusively. Of these 12 patients, 10 patients underwent testing involving *Mycobacterium tuberculosis* polymerase chain reaction (PCR), and the results were all negative, and Xpert was not used in all patients. Nine patients (75%) were actually treated with anti-TB medication after positive culture results for PBS.

**Table 1.** Demographic characteristics of patients with pulmonary tuberculosis who underwent bronchoscopy.

	Patients, *n* (%) (*N* = 92)
Age (years), mean ± SD	61.4 ± 14.2
Male gender	63 (69)
Initial clinical symptom or sign	
Fever	17 (19)
Cough or sputum	25 (35)
Body weight loss	4 (4)
Atypical image of chest X-ray	15 (16)
Underlying disease	
Human immunodeficiency virus	0/87^a^ (0)
Hematologic malignancy	7 (8)
Solid tumour	64 (70)
Bone marrow transplant	0 (0)
Solid organ transplant	3 (3)
Hemodialysis or peritoneal dialysis	7 (8)
Diabetes mellitus	24 (26)
Underlying condition	
Neutropenia (AN*C* < 500/m^3^)	0 (0)
Steroid use^b^	5 (5)
Immunosuppressant use^c^	7 (8)
TNF-alpha blocker	2 (2)
Cytotoxic chemotherapy within 1 month	14 (15)
MTB PCR	21/86^d^ (24)
Xpert MTB/RIF	6/11^d^ (55)

^a^HIV screening test was performed in 87 of 92 patients.

^b^Corticosteroid use is defined as the use of corticosteroids at a mean minimum dose of 0.3 mg/kg/day of prednisolone equivalent for ≥3 weeks.

^c^Treatment with immunosuppressants, e.g. T-cell inhibitor, during the previous 90 days.

^d^Ofthe total 92 patients, MTB PCR and Xpert MTB/RIF were used in 86 and 11 patients, respectively.

## Discussion

We demonstrated that PBS could improve the diagnostic yield by 20% (18/90 patients; 18 patients including 12 with positive mycobacterial culture results only from PBS and 6 with positive AFB smear only from PBS) when diagnosing pauci-bacillary pulmonary TB in immunocompromised patients. About 8% of the patients could be diagnosed rapidly because of AFB smear microscopy positivity from PBS, and 16% of the patients were confirmed to have pulmonary TB based on the culture results. Thus, these patients would have been missed if PBS had not been obtained.

Clinicians have difficulties to diagnose pulmonary TB, especially in immunocompromised patients, as they often present with atypical clinical manifestations of TB. Additionally, the current diagnostic tests for TB, such as AFB smear microscopy and Xpert, are insufficient to rule out pauci-bacillary pulmonary TB [11–13]. Delayed diagnosis and treatment of TB may contribute to transmission of TB and increase the severity and mortality of the disease [14]. Although the mycobacterial burden of pauci-bacillary pulmonary TB is lower than that of smear-positive cases, it has been reported that pauci-bacillary pulmonary TB accounted for about 17% of transmissions in a previous study [15]. Therefore, it is important to obtain appropriate respiratory specimens, as well as develop more sensitive diagnostic tests to improve the diagnostic yield.

PBS could be collected after bronchoscopy examinations easily. It has been reported that a no negligible portion of patients (about 9%–25%) were diagnosed with pulmonary TB based on AFB smear microscopy and mycobacterial culture of PBS exclusively in previous studies [3,4,16]. It is meaningful to diagnose additional patients with pulmonary TB rapidly based on AFB smear microscopy of PBS for public health and infection control. Additionally, identification of *M. tuberculosis* is crucial because clinicians can treat the patients based on susceptibility results of anti-TB medication. This could reduce the transmission risk of multidrug-resistant *M. tuberculosis* and prevent progression of the disease caused by delayed adequate treatment. In addition, immunocompromised patients are commonly taking several drugs such as immunosuppressant’s. Because these drugs would have drug-drug interactions with anti-TB medication, the results of drug susceptibility to anti-TB medication could provide opportunities for more appropriate and earlier anti-TB treatment in immunocompromised patients. The reason for the improved diagnostic yield of PBS has not been well known. This might be due to the possibility that pre-bronchoscopy sputum specimens were not adequate. Additionally, bronchoscopy examinations might irritate the mucosa of the airway and help patients to expectorate deep-seated sputum in sputum-scarce patients.

There are some limitations in this study. First, it was a retrospective study in a single large tertiary referral centre. Therefore, there could be patient selection bias. Second, we only included immunocompromised patients with pauci-bacillary TB. So, it is difficult to directly apply our findings to immunocompetent patients with pauci-bacillary TB, although there is no biologic plausibility to preclude the benefit of PBS. Third, we could not compare the diagnostic performances, such as sensitivity and specificity, of pre-bronchoscopy sputum, bronchoscopy specimens, and PBS. Fourth, we did not evaluate the additional value of Xpert and Xpert Ultra from PBS in this study because Xpert has been conducted in selected patients with suspected tuberculosis due to the national insurance coverage criteria for this test and Xpert Ultra could not be performed in our hospital. Further studies should be needed to evaluate the additional value of Xpert and Xpert Ultra from PBS to diagnose pauci-bacillary pulmonary TB. Finally, bronchoscopy examinations have limited feasibility, especially in high TB burden and low-income countries due to limited resources. Therefore, the results of this study have limited implications in these countries.

In conclusion, PBS should be recommended after bronchoscopy examinations in immunocompromised patients with suspected pauci-bacillary pulmonary TB. PBS use could be considered as a complementary approach for diagnosing pulmonary TB.

## Data Availability

The data that support the findings of this study are available from the corresponding author, Sung-Han Kim, upon reasonable request.
